# Fibrolamellar Hepatocellular Carcinoma and Noncirrhotic Hyperammonemic Encephalopathy

**DOI:** 10.1155/2018/7521986

**Published:** 2018-12-09

**Authors:** Oscar Suarez, María Perez, Martin Garzon, Rodrigo Daza, Geovanny Hernandez, Carolina Salinas, Jorge Ceballos, Enrique P. de Leon, Jacqueline Mugnier, Oscar Beltrán, Adriana Varón

**Affiliations:** ^1^Gastroenterology Fellowship, Universidad del Rosario, School of Medicine and Health Sciences, Bogotá, Colombia; ^2^Department of Medicine, Division of Internal Medicine, Universidad del Rosario, Bogotá D.C., Cundinamarca, Colombia; ^3^Department of Medicine, Division of Gastroenterology and Hepatology, Fundación Cardio Infantil, Bogotá D.C., Cundinamarca, Colombia; ^4^Department of Medicine, Division of Pathology, Fundación Cardio Infantil, Bogotá D.C., Cundinamarca, Colombia

## Abstract

Fibrolamellar hepatocarcinoma is an infrequent liver tumor, currently considered to be a variant different from hepatocarcinoma. The differences lie in genomic alterations, a greater prevalence of fibrolamellar hepatocarcinoma in young patients, and its lack of association with underlying liver disease. The clinical presentation is unspecific, with symptoms ranging from abdominal pain, malaise, and weight loss to atypical manifestation which include hyperammonemic encephalopathy. We present the case of a 33-year-old woman with no prior medical history who presented with a coma and a diagnosis of inoperable fibrolamellar hepatocarcinoma requiring a cadaver donor transplant. While she was on the waiting list, she received hemofiltration and ammonium benzoate treatment, with progressive improvement in her state of consciousness.

## 1. Introduction

Fibrolamellar hepatocellular carcinoma (FHCC) is a rare primary liver tumor which was described by Edmondson in 1956 [1]. It was initially thought to be a variant of hepatocellular carcinoma (HCC), but today it is considered to be a completely different entity. It is associated with few chromosomal changes and genomic heterogeneity, compared with HCC [2]. It occurs in the absence of cirrhosis or viral hepatitis [3].

Fibrolamellar hepatocellular carcinoma is a rare disease which infrequently requires transplantation [4]. It represents less than 1% of all primary liver cancers [5] and is recognized more as a pediatric and adolescent disease [6, 7]. However, some studies have found two incidence peaks at 10 to 30 years and 70 to 79 years of age [8]. In the genomic analysis of this tumor, three molecular types have been found: the proliferative type, characterized by altered expression of the genes which regulate the proliferation and activation of mTOR signals; the inflammatory type, with alteration in the genes which regulate inflammation and the production of cytokines; and the “unlisted” type in which no relationship is found between its genetic expression and liver tumors. Neuroendocrine-regulating genes are found in all three types of tumors [9]; this genomic print suggests that these tumors may have a different origin than that of hepatocarcinomas, which could produce directed treatments in the future [10].

## 2. Case Report

A 33-year-old female patient consulted due to a two-week history of disorientation and somnolence which progressed to stupor, requiring invasive mechanical ventilation. The patient had a history of a Cesarean section four months prior to her admission to the emergency room, without complications during pregnancy or delivery, and no known medical, pharmacological, allergic, or family history. The physical exam was remarkable for the presence of jaundice and hepatomegaly, without clinical signs suggesting cirrhosis.

**Figure 1:**
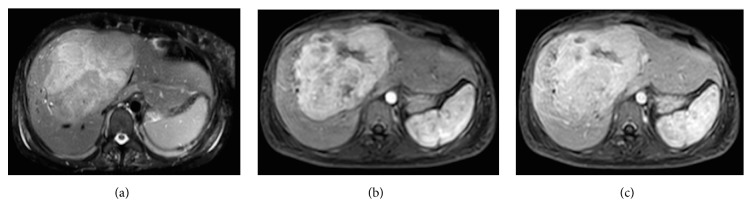
Abdominal magnetic resonance images: (a) T1W, (b) arterial phase, and (c) portal phase. Focal hepatic lesion with lobulated borders and a 13 cms of diameter which has signal heterogeneity in T1 and T2 sequences, with restrictive regions in the diffusion sequence; the lesions are highlighted in the arterial phase; they are practically isointense in the portal and late phase. Irregular hypointense linear area which corresponds to a scar zone.

The admission laboratory tests registered an altered liver profile (Table 1) with elevated ammonia (595.7 umol/L). Therefore, an abdominal tomography was performed, reporting a focal liver lesion which was interpreted to be a hepatic adenoma. Abdominal magnetic resonance imaging was then carried out (Figure 1) which showed results compatible with fibrolamellar hepatocarcinoma, with no signs of cirrhosis or portal hypertension.

In light of her neurological deterioration, a simple head tomography was performed which showed unspecific periventricular lesions. A lumbar puncture ruled out infectious involvement, and telemetry reported moderate encephalopathy without seizure activity.

Operating under the diagnostic impression of hyperammonemic encephalopathy, urea metabolism disorders were studied, including urinary orotic acid levels, serum amino acids, and serum citrulline levels, all of which were normal (Table 2).

**Figure 2:**
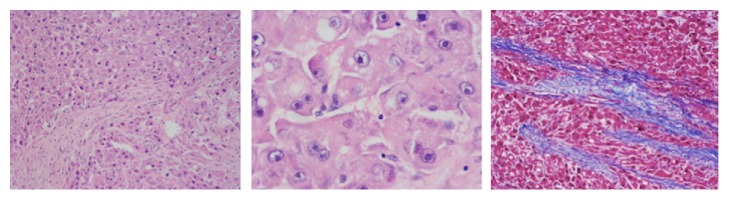
Histopathological findings of the tumor. Tumor made up of polygonal cells with a broad eosinophilic cytoplasm and a vesicular nucleus with a prominent central nucleolus. The tumor cells are separated by fibrocollagenous lamellae which are highlighted with Masson's trichrome staining.

Given the lack of an etiology of the hyperammonemic encephalopathy and findings suggestive of fibrolamellar hepatocellular carcinoma, a liver biopsy was performed which confirmed the diagnosis through immunohistochemistry (Figure 2) which reports diffuse and intense expression for cytokeratin 7Y CD68, with marked sinusoidal capillarization with CD34 and expression for CD68 there is weak but diffuse expression for glutamine synthetase, and glypican 3 and amyloid A are negative. The expression for B-catenin is membrane, without aberrant expression. The cell proliferation index determined with KI 67 is 5%. This morphological and phenotypic pattern favors a fibrolamellar variant hepatocellular carcinoma.

Extension studies ruled out metastatic bone or chest involvement, and neoplastic lesions elsewhere. The hyperammonemic encephalopathy was associated with intrahepatic shunts secondary to the tumor. Management was begun with continuous veno-venous hemofiltration and ammonium benzoate treatment, which led to a progressive improvement in her state of consciousness.

On surgical evaluation, resection of the lesion was contraindicated due to extrinsic vascular and bile duct compression. Therefore, cadaveric donor liver transplantation was performed, with no postoperative complications. The explant study evidences the presence of a single lesion confined to the liver corresponding to the FHCC, with borders of hepatic hilum and suprahepatic section free of tumor, not identifying vascular invasion, perineural, lymphatic, nor lymph node, so resection of regional lymph nodes was not necessary.

The patient is currently being followed by the hepatology department, with no signs of neurological alterations or tumor relapse, and a normalized liver profile (Figure 1).

## 3. Discussion

### 3.1. Clinical Presentation

The presenting symptoms include abdominal pain, weight loss, and malaise, and hepatomegaly may be found on physical exam [3, 11]. The following have been described as atypical manifestations: vascular abnormalities such as compression of the vena cava [12] or Budd–Chiari syndrome [13]; amyloid deposition [14]; biliary obstruction due to bile duct invasion [15]; and presentations such as bone [16], pancreas [17], and ovarian [18] metastases.

### 3.2. Laboratory Findings

Transaminases and alkaline phosphatase are normal or slightly elevated. If the patient should present with markedly elevated alkaline phosphatase, bile duct growth or obstruction should be suspected [11]. Serum alpha fetoprotein (AFP), which is usually elevated in HCC, is within normal limits, or slightly elevated in FHCC [11]. Other markers such as vitamin B12-binding protein, or haptocorrin, and neurotensin may be elevated [19, 20]. It has been suggested that haptocorrin levels are associated with disease progression [20].

### 3.3. Images

On ultrasound, it is characterized by being a well-defined mass with heterogeneous echogenicity [11]. On tomography, it is seen as a lobulated, heterogeneous mass; it may be hypoattenuating with calcifications (43% of cases) and a central scar (46% of cases) [21]. Necrosis without intratumor hemorrhaging may also be found. In the arterial phase, it is hyperattenuating due to hypervascular tumor cells with hypovascular bands around them. In the portal phase, they may be isoattenuating, but up to 40% may be hyperattenuating and 20% hypoattenuating [22].

On magnetic resonance, they are seen as hypointense tumors in T1W and hyperintense tumors in T2W, also with a central scar which is hypointense in both images. In the arterial phase with gadolinium, there is heterogeneous capture which washes out, showing a hypointense lesion in the portal venous phase [7, 23].

### 3.4. Hyperammonemic Encephalopathy

Noncirrhotic hyperammonemic encephalopathy is a potentially lethal complication in patients with rapid growth liver tumors. There have been reported cases of hyperammonemia related to fibrolamellar carcinoma associated with intrahepatic shunts of nitrogenated compounds, due to the fact that the necrotic and neoplastic cells are unable to filter out this type of compounds [24, 25].

It has also been proposed that FHCC may cause the release of an OCT (ornithine transcarbamylase) enzyme inhibitor or that there may be increased ornithine decarboxylase activity [26, 27]. This type of encephalopathy may also be triggered in patients with fibrolamellar carcinoma who are receiving chemotherapy, which causes increased tumor lysis [27].

There is no protocol for the management of hyperammonemic encephalopathy. Chapuy et al. [27] have proposed a diagnostic and treatment algorithm which includes measuring ammonium levels in patients with FHCC, regardless of liver function. If the levels are elevated, the next step is to evaluate urea cycle dysfunction by measuring urinary and serum amino acids and urinary organic acids and analyzing orotic acid.

Treatment is based on decreasing the nitrogen load, removing excess ammonia, and correcting the precipitating causes. Fluids with dextrose should be started to control dehydration and catabolism. An increase of 1.5 times the basal requirement is proposed as the caloric support goal: 60 kcal/kg/day in adults [27]. Treatment with sodium benzoate and sodium phenylacetate, used in urea cycle disorders, is recommended if the ammonium levels are greater than 100 uM [27]. If this treatment fails, or if the levels are over 500 uM, hemodialysis should be started.

Another consideration for the use of dialysis is the unavailability of ammonia removers.

### 3.5. Prognosis and Treatment of FHCC

Surgical resection is the mainstay of treatment, since it represents a curative option. Complete resection with negative margins and node dissection is the goal of this treatment. In the systematic review carried out by Mavros et al. [28], partial hepatectomy represented a 5-year survival rate of 70%, while liver transplantation had a 34% survival rate.

Comparing any surgical treatment with not undergoing surgery yielded a 5-year survival rate of 44% versus 0% [28]. Even patients in advanced stages are benefited by surgical treatment [29]; in stage IV patients, the 5-year survival rate is 66%, and 47% at 10 years [30]. It is important to point out that, in the systematic review referred to above, 90% of the liver transplants were performed before 1990. By 2013, 63 transplants had been performed in the United States due to FHCC; of this population, 57.1% were females. At six months, acute rejection was reported in 4.8% of the patients, 1.6% required retransplantation, and tumor recurrence was reported in 9.5% of transplanted patients. Five-year disease-free survival was 46%. The factors associated with graft loss included bile duct complications, infection, and disease recurrence [31].

Recurrence following complete surgical resection ranges from 33 to 100%, with a median of 10 to 33 months [29]; recurrence does not exclude the indication for a new resection, which has been associated with a 48% improvement in 5-year survival [2]. The factors associated with recurrence following liver transplantation include regional node involvement, metastasis, and stage IVB [30].

The role of adjuvant and neoadjuvant chemotherapy is still unclear, as there are no prospective studies to evaluate the different chemotherapeutic lines in FHCC. In some reports, it has been associated with a greater survival [32].

A comparison of the prognosis of patients with fibrolamellar carcinoma to that of patients with hepatocarcinoma shows improved survival (RR 2.02 95% CI 1.38 – 3.16) [33]. Cirrhosis is a poor prognostic factor in hepatocarcinoma; a subgroup analysis limited to patients without cirrhosis found no significant difference in five-year survival (RR 1.69 95% CI 0.69 – 4.17) [33]. Thus, the prognosis of fibrolamellar carcinoma is similar to that of hepatocarcinoma in patients without cirrhosis and better than that of hepatocarcinoma in patients with cirrhosis (*β*=−5.62, 95% CI −2.11 to −7.14 p<0.001) [33].

Our patient had FHCC with noncirrhotic hyperammonemic encephalopathy secondary to intralesional shunts, which required ongoing hemodialysis to control the encephalopathy. She had a large, inoperable lesion with no evidence of secondary extrahepatic involvement.

Therefore, she underwent a successful liver transplantation with complete resolution of the encephalopathy, no need for dialysis, normalization of her ammonia levels, and adequate progress at four months' follow-up with immunosuppressant management. A case was found in the literature of a 23-year-old patient with coma secondary to hyperammonemia, who was also diagnosed with FHCC with no evidence of metastatic lesions, underwent transplantation with postoperative initiation of sorafenib, and was reported as disease-free after one year of follow-up [34].

## 4. Conclusion

Fibrolamellar hepatocellular carcinoma may produce noncirrhotic hyperammonemic encephalopathy due to enzyme deficits or secondary to portosystemic shunts. The treatment of choice is surgical resection, when possible; and, in selected patients, liver transplantation may be a definitive treatment option with adequate short-term results.

## Figures and Tables

**Table 1:** Patient's laboratory results on admission and four months after transplant.

**Laboratory**	**Admission**	**4 months after transplant**
Leucocytes	10,940 cells/ul	4,880 cells/ul

Neutrophils	8,300 cells/ul	2,718 cells/ul

Lymphocytes	1,860 cells/ul	1,747 cells/ul

Hemoglobin	15 gr/dl	12 gr/dl

Hematocrit	32.6%	37.3%

Platelets	544,000 cells/ul	366,000 cells/ul

INR	1.2	1.1

Total bilirubin	2.73 mg/dl	0.7 mg/L

Direct bilirubin	2.34 mg/dL	0.4 mg/dL

Indirect bilirubin	0.39 mg/dL	0.3 mg/dl

Alkaline phosphatase	1,007 U/L	371 U/L

GGT	1,636 U/L	501 U/L

ALAT	261 U/L	39 U/L

ASAT	264 U/L	21 U/L

Glycemia	137mg/dL	85mg/dL

Ammonium	595.7 umol/L	14 umol/L

Creatinine	0.51 mg/dL	0.7 mg/dL

BUN	18.2 mg/dL	22mg/dL

AFP	1.5ng/ml	

**Table 2:** Urea cycle metabolites.

Laboratory	Patient's value	Normal value
Pyruvic acid	0.11 mmol/L	0.03 – 0.12 mmol/L

L-lactate lactic acid	1.23 mmol/L	0.5 – 2.2 mmol/L

Lactate/pyruvate ratio	11.18	0- 25

Phosphoserine	11 umol/L	1 to 17 umol/L

Taurine	47 umol/L	11- 202 umol/L

Phosphoethanolamine	0 umol/L	0 – 189 umol/L

Aspartic acid	7 umol/L	0- 31 umol/L

Threonine	33 umol/L	45 – 194 umol/L

Serine	44 umol/L	73 – 154 umol/L

Asparagine	46 umol/L	32 – 98 umol/L

Glutamic acid	141 umol/L	34 – 177 umol/L

Glutamine	399 umol/L	204 – 1101 umol/L

Glycine	95 umol/L	94 – 553 umol/L

Alanine	145 umol/L	161 – 535 umol/L

Citrulline	9 umol/L	1 – 33 umol/L

Ornithine	68 umol/L	16 – 129 umol/L

Arginine	18 umol/L	13 – 128 umol/L

Lysine	63 umol/L	35 – 220 umol/L

Anti-citrulline antibodies	< 7 IU/ml	0 – 16 IU/ml

Urinary orotic acid	2.3 mmol/mmol creatinine	0.4 – 5.1 mmol/creatinine

## Abbreviations

FHCC: Fibrolamellar hepatocellular carcinoma
HCC: Hepatocellular carcinoma
AFP: Serum alpha fetoprotein
OCT: Ornithine transcarbamylase.
